# Interaction and medical inducement between pharmaceutical representatives and physicians: a meta-synthesis

**DOI:** 10.1186/s40545-016-0089-z

**Published:** 2016-11-17

**Authors:** Shahrzad Salmasi, Long Chiau Ming, Tahir Mehmood Khan

**Affiliations:** 1 Collaboration for Outcomes Research and Evaluation (CORE), Faculty of Pharmaceutical Sciences, The University of British Columbia, Vancouver, British Columbia Canada; 2 Unit for Medication Outcomes Research and Education (UMORE), Pharmacy, School of Medicine, University of Tasmania, Hobart, Tasmania Australia; 3 School of Pharmacy, Monash University, Sunway City, Selangor Malaysia; 4 Department of Pharmacy, Abasyn University, Peshawar, Pakistan

**Keywords:** Inducement, Motivation, Health care quality, Pharmaceutical marketing, Prescription behavior, Marketing ethics, Pharmaceutical representatives

## Abstract

**Background:**

It has been proven that the interaction between pharmaceutical representatives and physicians can directly influence the latter’s prescribing behaviour. This meta-synthesis aims to explore the available studies regarding the nature of the interaction that takes place between pharmaceutical representatives and physicians. It highlights the different aspects of that interaction by investigating the reasons why these meetings happen in the first place, their benefits and drawbacks and their impact on patients’ health and, ultimately, the health of the public.

**Methods:**

A search for published articles was conducted in April 2015. Three databases (PubMed, Ovid Medline, and ProQuest) were searched for articles published between January 2000 and April 2015. Authors worked autonomously and in pairs to select eligible articles. In this case, the meta-synthesis approach was used to develop a fuller understanding and to facilitate new knowledge by bringing together qualitative findings on physician-PR interaction. ‘Meta-synthesis’ is the process of amalgamation of a group of similar studies with the aim of developing an explanation for their findings (Walsh and Downe, J Advanc Nurs 50: 204–211, 2005). A thematic content analysis was conducted on the 15 included full text articles (qualitative and quantitative studies) whereby the original authors’ understanding of key concepts in each study was identified and listed in a summary form in the data extraction sheet under “key findings” column. These findings were then juxtaposed to identify homogeneity and dissonance (Walsh and Downe, J Advanc Nurs 50: 204–211, 2005). Homogenous findings were then coded together on a different data extraction table to form a theme.

**Results:**

A total of 15 articles met the inclusion criteria and were included in this meta-synthesis;six from the United States, two from Libya, and one each from Turkey, Peru, India, Germany, the United Kingdom, Yemen, and Japan. Six main themes were derived from the included articles: 1-the frequency of pharmaceutical representatives’ visits, 2-the perceived ethical acceptability of the interactions between pharmaceutical representatives and physicians, 3-the attitudes held by physicians towards visits by pharmaceutical representatives, 4-their perception of the effect of such visits on prescription patterns, 5-reasons to accept or reject pharmaceutical representatives, and lastly, 6-guidelines.

**Conclusions:**

The physicians referred to pharmaceutical representatives as efficient and convenient information resources and were willing to meet them and accept their gifts. It was also evident that most physicians believed that their prescribing would not be influenced by pharmaceutical representatives.

**Electronic supplementary material:**

The online version of this article (doi:10.1186/s40545-016-0089-z) contains supplementary material, which is available to authorized users.

## Background

The pharmaceutical industry significantly influences the economy and healthcare system of a country. According to The World Health Organization(WHO), by 2017,the global pharmaceuticals market will be worth approximately US$400billion with one third of thesales revenue of pharmaceutical companies spent on marketing their products [1]. Physicians are the prime target of pharmaceutical marketing teams, in which pharmaceutical representatives (PRs) play a crucial role [1]. The job of the PRs is to visit physicians to promote their company’s products [2, 3]. In addition, free physician samples, gifts, company supported conferences, workshops and events are also brought to the notice of physicians through PRs. Among all the promotional expenses, detailing is the largest category; estimated US$20.4 billion has been used for this purpose in United States (US) in 2015 [4, 5].

Given the above, PR-physician interactions have the potential to result in a conflict of interest whereby physicians might fail to abide by their moral, legal and professional obligations for personal gain [6, 7]. Research has shown that the interaction between PRs and the physicians can directly influence the latter’s prescribing behaviour; it has been observed that the rate of prescriptions increases after physicians see a PR or accept free samples [8]. This has led to increasing concern over irrational and inappropriate prescribing practices [9] which could jeopardize patient care, harm the patients, promote the misuse of drugs, increase costs to the healthcare system, distort public opinion of the healthcare industry and erode the trust of the patients in the integrity of medical decision making [8, 10].

Wazana et al., published a review in 2000 that identified the extent of and attitudes toward the interplay of relationship and its impact between physicians towards the PR [11]. Wazana’s review received a mixed response from the health care communities because he proposed that the extent of physician-PR interactions could potentially affect prescribing and professional behaviour [12–18]. Ten years later, Spurling et al. who performed a systematic review on 58 articles, reported that exposure to information from pharmaceutical companies was associated with either lower prescribing quality or increase in prescribing frequency or no association was detected. Finally, with only one exception among the 58 studies included in the review by Spurling et al.; exposure to information from PRs was associated with an increase in prescribing costs or no association was detected. They reported that interaction of physicians with PRs does not negatively affect prescribing [3]. This is the first meta-synthesis that fills the literature gap on the interaction between PRs and physician. The purpose of this meta-synthesis, therefore, is to explore the available studies regarding PR-physician interactions especially to highlight the detailing aspects of such interactions from the physician’s point of view.

## Methods

A systematic literature search of computerized databases was conducted in April 2015 using PubMed, Ovid Medline and ProQuest. A meta-synthesis approach was used to integrate results from various studies in order to give a comprehensive overview of the interaction that takes place between pharmaceutical representatives and physicians [19]. Research papers published between January 2000 and April 2015 which presented evidence concerning such interactions, were included. The search strings used can be found in Supporting Information Additional file 1: Table S1.

The search strings used were very specific. This is because we were not interested in marketing methods other than detailing. Our purpose was to focus on the interaction that takes place between PRs (and not any other promoter/ representatives from other industries who might meet with physicians) and physicians (and not any other prescriber such as prescribing nurses, dentists, etc.). Hence the search strings were chosen carefully to retrieve the most relevant studies. To ensure all relevant papers were included, we searched the references of the studies that were included. Furthermore, Monash integrated search panel for Science Citation Index were also used to screen the relevant citations of the included studies.

A total of 218 papers were identified using the search strings outlined in Additional file 1: Table S1. Duplicates and articles published before the year 2000 were excluded. The title and abstract of the 72 remaining papers were assessed for eligibility against the inclusion criteria. This was performed independently by two reviewers, who classified the papers into three groups of “to include”, “to exclude” and “unclear”. Both reviewers (SS and TMK) met to compare their independent reviews. A paper would be included or excluded once agreed by both reviewers. In the case of disagreement, or when either reviewer was unsure, the opinion of a third person (LCM) was sought.

The inclusion criteria were articles focusing on detailing and physician interaction with PRs. Studies related to marketing methods other than detailing, such as direct to consumer advertising and ghost writing were excluded. Papers written in languages other than English were excluded as we did not have the necessary skills to interpret them. We also excluded studies focused on prescribers other than physicians, such as prescribing nurses. Opinion papers and interventional studies were also excluded.

**Fig. 1:**
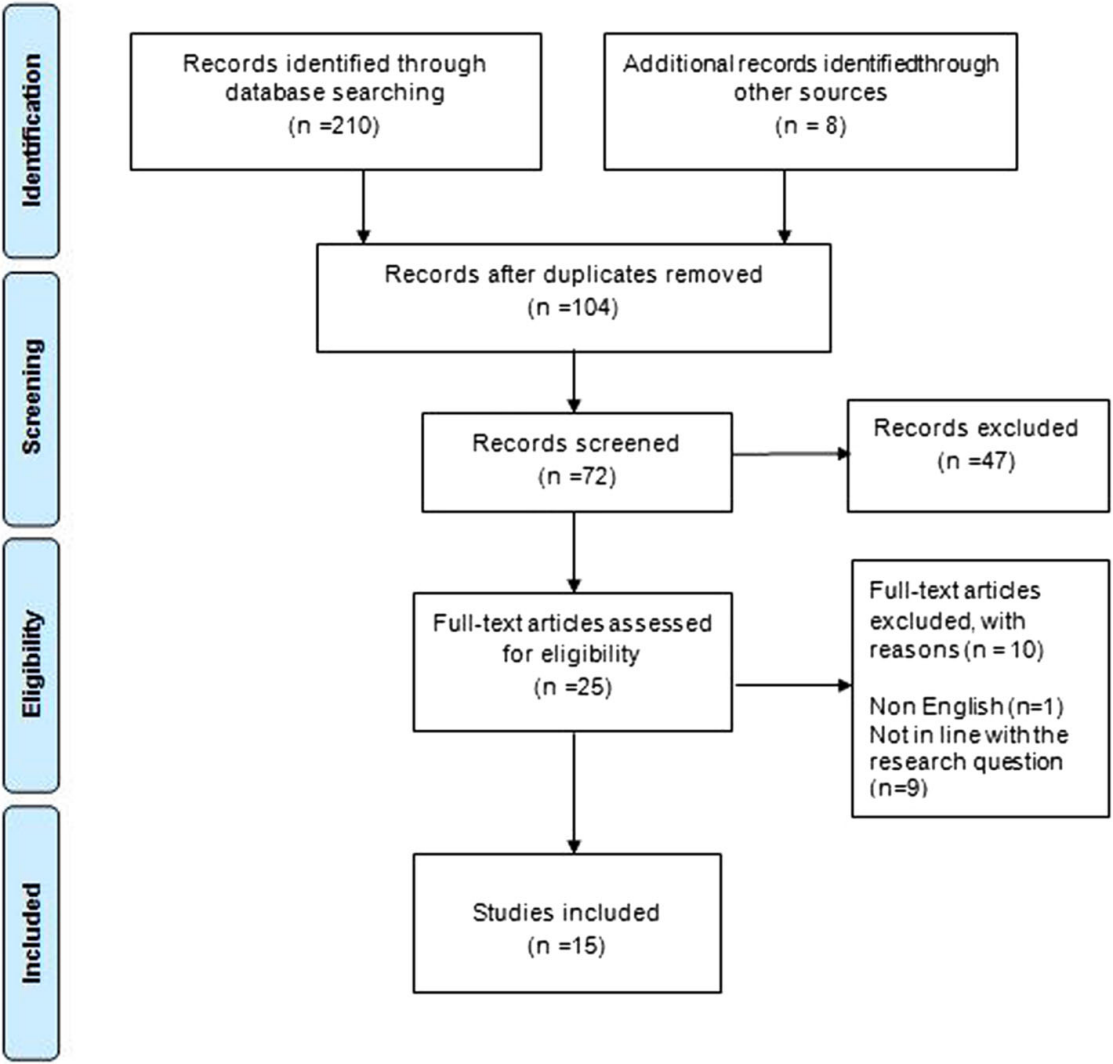
PRISMA diagram demonstrating the search strategy and its results

The full text of 25 papers was read by the first author. A number of studies were identified at this stage as not being written in English (i.e. their title and abstract were written in English but the full-text was not) and these were therefore excluded, along with a number of others that were found to be irrelevant. This left 15 papers. Details about the number of the included and excluded articles are shown in Fig. 1. The Preferred Reporting Items for Systematic Reviews and Meta-Analyses (PRISMA) checklist is presented in Additional file 2: Table S2.

### Analysis and theme synthesis

‘Qualitative meta-synthesis’ is the process of amalgamation of a group of similar qualitative studies with the aim of developing an explanation for their findings [20]. In this case, the meta-synthesis approach was used to develop a fuller understanding and to facilitate new knowledge by bringing together qualitative findings on physician-PR interaction. The 15 included papers were subjected to data extraction using a pre-designed data-extraction Excel worksheet on Microsoft Office® 2014. A thematic content analysis was conducted on the15 included full text articles whereby the original authors’ understanding of key concepts in each study was identified and listed in a summary form in the data extraction sheet under “key findings” column. These findings were then juxtaposed to identify homogeneity and dissonance [20]. Homogenous findings were then coded together on a different data extraction table to form a theme. Six broad themes were formulated for the results section; 1-The frequency of pharmaceutical representatives’ visits, 2-The perceived ethical acceptability of the PR-physician interactions, 3-Physicians’ attitudes towards PR visits, 4-Physicians’perceptions of the effect of PR visits on prescription patterns, 5-Reasons to accept/reject PRs and 6-Guidelines. Findings were scrutinized under each theme to see if further sub-categories were warranted. The arguments for and against PR interaction with physicians were very rich. We were hence unable to take account of all the arguments, in great detail. Moreover, while all the included studies presented information about PR-physician interactions, they reported different aspects of such interactions since they examined the interaction from different angles, hence a thematic analysis was chosen to classify and summarize the findings in a systematic, easy to understand manner. Through the six themes formulated, we hope to provide an overview of the main aspects of the PR-physician interactions identified.

## Results

A total of 15 publications were included in the study covering studies undertaken in a range of countries; six from the US, two from Libya, one each from Turkey, Peru, India, Germany, the United Kingdom (UK), Yemen and Japan. Eight of these articles focused on physicians’ attitudes and views regarding physician-PR interactions [9, 21–27]; four focused on the nature of the interaction [26, 28–30]; two concentrated on the frequency of these interactions [31, 32]; and two focused on the reasons behind such interactions [33, 34]. The detailed themes and extracted text is presented in Additional file 3. Study and participants’ characteristics can be found in Table 1.

**Table 1:** Characteristics of the included studies

Study	Participant’s Specialty (N)	Gender M/F	Years of Practice (N)	Practice Setting	Country
Morgan et al. 2006 [25]	Gynecologists (397)	125/92	NA	Obstetrics-gynaecology partnership or group (44.2%) Solo practice (27%) Multispecialty group (14.9%) University full-time faculty and practice (10.7%) Health maintenance organisation (3.3%)	US
Anderson et al. 2009 [28]	Gynecologists (251)	143/108	Mean (SD): 22 (11)	Private practice (178) Community hospital (25) University hospital (30) Other (16)	US
Wang et al. 2009 [27]	Ophthalmology trainees (122): 1^st^ year residents (32) 2^nd^ year residents (44) 3^rd^ year residents (28) Fellows (17) Unknown (1)	NA	NA	NA	US
Misra et al. 2010 [24]	Psychiatry trainees (17): Residents (12) Fellow trainee (5) Psychiatry faculty (58): Assistant professor (21) Associate professor (6) Professors (13)	NA	NA	Academic medical centre	US
Fischer et al. 2009 [34]	Internal medicine (29) Family medicine (17) Pediatric medicine (2) Geriatric medicine (3)	23/38	15	Academic affiliation (21) Community-based practice (24) Academic affiliation & community-based practice (8) Other (8)	US
Brett et al. 2003 [23]	Residents (39) Faculty physicians (37)	49/27	NA	Medical school	US
Sarikaya et al. 2009 [32]	Medical students 2nd year students (280) 3rd year medical students (308)	168/140	NA	Marmara University School of Medicine (MUSM) Ege University School of Medicine (EUSM)	Turkey
Saito et al. 2010 [26]	Internal medicine (214) General surgery (181) Orthopedic surgery (177) Pediatrics (221) Obstetrics (210) Psychiatry (197) Ophthalmology (209)	1084/326	<10 (339) 11–20 (488) 21–30 (428) >31 (155)	Office (822) Hospital (588)	Japan
Roy et al. 2003	Senior executives in drug companies (15) PRs (36) Physicians (25) Chemists (25)				India
Alssageer et al. 2013 [31]	General Practitioner (GP) (274) Surgeon (99) Resident MO (41) Anesthesiologist (61) Specialist (91) Others (42)	371/237	1-3 (288) 4–6 (82) 7–9 (45) >10 (193)	Public (512) Private (34) Both (62)	Libya
Alssageer et al. 2012 [22]	GP (274) Surgeon (99) Resident MO (41) Anesthesiologist (61) Specialist (91) Others (42)	371/237	1-3 (288) 4–6 (82) 7–9 (45) >10 (193)	Public (512) Private (34) Both (62)	Libya
Prosser et al. 2003 [33]	GP (107)	76/31	<10 (6) 11–20 (55) >20 (46)	Health authorities in the North West of England (107)	UK
Al-Areef et al. 2013 [21]	Physicians (32) (interns, GPs/medical officers, residents and specialists)	4/28	NA	NA	Yemen
Lieb et al. 2010 [29]	Neurologists/psychiatrists (83) Primary care physicians (76) cardiologist (49)	104/98 Unknown gender (6)	NA	NA	Germany
De Ferrari et al. 2014 [9]	Internal medicine (59) General surgery (32) Pediatrics (28) Anesthesiologist (13) Obstetrics (16)	96/52	NA	Peruvian public general hospital.	Peru

*Abbreviations*: *GP* General Practitioner, *NA* not applicable/available, *UK* United Kingdom, *US* United States of America, *MO* Medical Officer, *SD* Standard deviation

### Theme 1.0: The Frequency of PRs visits

The first step in investigating and understanding the interaction between the PRs and the prescribers is to determine how frequent such interactions are, and if they are significant enough.

Research has shown that frequency of PR visits to physicians- skilled health-care professionals trained and licensed to practice medicine- is considered to be a good indicator of an active relationship between physicians and the pharmaceutical industry. Lieb et al. reported that 77.0% (n = 160) of German respondents met with PRs at least once a week, and 19.0% (n = 39) were visited by PRs on a daily basis [29]. Other studies also reported that PRs visit physicians regularly in order to promote their products [9, 21, 22, 26, 29, 32]. De Ferrari et al. studied the relationship between the specialty of the prescriber and the frequency of PR visits [9]. Physicians involved in teaching and those practicing paediatric medicine, were reported to receive more frequent visits, while anaesthetists were found to be least frequently visited by PRs [9]. Two studies reported that PRs visited physicians working in a private setting more frequently, while physicians working in community and university hospitals experienced less frequent visits [22, 23].

### Theme 2.0: Perceived ethical acceptability of the PR- physician interactions

The job of PRs is to introduce and market their company’s products. PRs ensure that physicians are informed of the benefits of the products and are therefore willing to prescribe them to their patients. The interaction between PRs and physicians entails a number of marketing methods such as gifts, sponsorships, free drug samples and free lunches. Moreover, in recent years, simple brand reminders such as stationery with the company’s logo and product name on them have increasingly been replaced by gifts of greater value, ranging from jewellery to iPads [30]. According to policymakers, such gifts have the potential to act as an ethical inducement and negatively impact prescribing behaviour and, ultimately, patient health [11, 30]. Brett et al., for example, conducted a survey in South Carolina where physicians were asked to rate 18 scenarios of interactions between physicians and PRs from the most ethically appropriate (score = 0) to the least ethically appropriate (score = 5). Results showed that physicians made distinctions about the ethical appropriateness of gifts of different value and different type; recreational gifts were rated to be significantly less ethically appropriate than educational gifts, as were the more expensive gifts [23]. According to De Ferrari et al., activities perceived to be most ethical were the provision of medical samples (81.8%) and continuing medical education (68.9%) [9, 25]. Roy et al. further added that, in India, some physicians justify gifts as compensation for the time spent listening to the PR, time which could have been spent on a patient [30].

*“PRs never try to bribe to sell their drug. (Gifts) are just a gesture to say thanks for the time the doctor gives. Let’s say a doctor sees three patients in 15 minutes, the PR us costing him those three patients in his 15-minute talk. So the PR tries to compensate with gifts since obviously he can’t compensate in cash”* [30].

In contrast, there were others who felt that air conditioners, washing machines, microwaves, cameras, televisions and expensive crystals were acceptable gifts [30].

### Theme 3.0: Physicians’ attitudes towards PR visits

The physician’s attitude is what determines whether they would be inclined to believe the information provided by the PR. Qualitative data demonstrating the attitude of physicians are presented in Additional file 4: Table S3.

#### 3.1 Perceived legitimacy of the PR

Legitimacy refers to the accuracy and accountability of the PRs as a source of information. According to Prosser et al., a relationship has been observed between the frequency of visits and the perceived legitimacy of the information provided: the more frequently visited physicians were more likely to consider the information provided to be of “high quality” [33].

Physicians have been reported, in a number of studies, to consider the information provided by the PRs as *“not trustworthy”, “very variable*” and *“depending on which PR you see*”; they also disclosed that the PRs usually choose the promotion of their company’s product over the actual benefits for the patient by hardly ever mentioning the drug interactions and the side-effects [9, 21, 29, 30, 33].

On the other hand, some physicians from a number of different studies recognized the professional authority of PRs as information providers and expressed satisfaction with the information provided. Just under half of all the participants (47.6%) in a study in Peru, for example, stated that the information provided by PRs helps them “learn about new products” and “stay up-to-date”. Similarly, Roy et al. reports that physicians were satisfied with the information provided despite the fact that side-effects were hardly ever mentioned [30]. De Ferrari et al. also reported that 24% of faculty (n = 10) and 18% of psychiatry residents (n = 3) believed that PRs provide useful and accurate information on new drugs [9].

Overall, while opinions on this matter differ among physicians, it can be concluded that physicians consider the information provided by PRs, factual but, to an extent, biased.

#### 3.2 Perceived benefits of the interaction with PRs

The perceived benefits of the interaction are the reason why physicians continue to see PRs, despite knowing that the information provided by them may, in some cases, be biased. These perceived benefits are explained below.

##### 3.2.1 Easy access to information

Research indicates that physicians considered the convenience of acquiring information from PRs, regarding both old and new drugs, to be the main benefit of meeting with them, This is true regardless of the country the physicians practiced in [21, 23, 24, 26, 29, 33, 34].

Physicians also reported that PRs are useful for obtaining research papers and journal off-prints. Physicians’ busy schedules leave them no time to look for evidence. Thus, representatives are seen as ‘short-cuts’ and “valuable sources of information”, simplifying the acquisition and evaluation of new product information [33].

“*I’m sure you could manage if you didn’t see another drug rep and I’m sure you could get the information if you wanted to, it’s just that it’s not that accessible, and it’s also whether you would have the time to actually sit and read it”* [33].

“*Although you try and keep up with journals and such like that, some things go by, you do miss things. So I feel like I’m keeping up to date a little bit. If I didn’t see reps I feel that I would be slightly disadvantaged in terms of my awareness of medications coming through*” [33].

*“[It is positive] that they can inform us about new products […] being launched in the market for the first time. Secondly, we can [hear about] alternatives from other companies that have the same effectiveness, low cost and less side effects*” [21].

The user-friendly and face-to-face nature of the interactions permits physicians to ask questions, and get instant answers;

“ *I think the answer is it’s user friendly, it’s very user friendly and its easy listening, you know, with your coffee listening to what they’ve got to say*” [33].

Furthermore, according to Prosser et al. [33], several physicians have commented that they remember the information better when communicated verbally;

“ *I can remember the information better after having talked to them*” [33].

“*Seeing a representative face to face tends to make a more lasting impression than reading*” [33].

##### 3.2.2 Free gifts and drug samples

The other perceived benefit of meeting PRs is the gifts and free samples provided by them. Such gifts can range from materials that are supposed to directly help the patients (blood sugar diaries, educational materials) to personalized gifts for the doctor; from cheap stationery to expensive so-called gifts that act as inducements [21, 34]. According to Alssageer et al.(n = 423) 86% of respondents reported to have received printed material (n = 480; 79%), simple gifts (stationery, n = 442; 73%) or drug samples (n = 418; 69%) at least once during the last twelve months [31].

“*Sometimes we need representatives in providing some medicines that we need it, some books or bulletin. Really, they help us in getting books, CDs and lectures from abroad that provided by some companies. They support us on this side a lot*” [21].

Free medication samples are the most frequently accepted gifts in the US and Germany. Physicians consider these gifts to be the most ethically acceptable and a great advantage of meeting with PRs [9, 29, 34].

Physicians and PRs justify such gifts on the grounds that free medications can be used to help patients who are unable to afford medications due to financial constraints. Free samples can also be used to determine the dose and side-effects before the patient has to invest in them [34, 36].

“*We want to make people happy and you make people happy often when you give them a sample”* [34].

Morgan et al. reported that the main reason for which the free drug samples are distributed is patients’ financial needs, followed by patients’ convenience and to build a good relationship with the patient, but less than two-thirds of physicians distributed free samples with a good basis of knowledge as to the efficacy of the sample product [25].

##### 3.2.3 Social aspects of the interaction

Some physicians liked the casual, friendly aspect of the interaction and thought of the representatives as more of their friend than a promoter;

*“Going out to dinner as a group….That’s why we do it, more of a social setting outside of the wards”* [34].

#### 3.3 Perceived drawbacks of the interaction with PRs

Despite the above-mentioned positive attitudes towards PRs, the prescribers do admit that there are certain drawbacks to seeing PRs. These drawbacks, in their opinion, are:

##### 3.3.1 Negative impact on the patient

Some physicians believe that PRs take up a proportion of the doctor’s time, which could have been spent attending to patients [29, 30].

*“Doctors always perceive MRs’ visits as an intrusion. Every minute taken up by the MR is time which could have been spent seeing patients and making money in the clinic. Often, MRs queue up early in the morning for doctors who allow only the first three MRs to see them”* [30].

Al-Areefi et al. reported that some physicians hate the fact that PRs interrupt their patient care process, especially those working in the emergency departments with a high workload and a large number of patients to attend to. These physicians have absolutely no time to listen to PRs and even refer to them as “time wasters” [21].

Some other physicians reported discomfort with or dislike of the interactions, and this appeared to be rooted in their perception that PRs harm the ethical reputation of the profession and adversely affect patients [21, 34].

*“My rule is I [listen but] don’t believe anything they’re saying”* [34].

##### 3.3.2 Pressure from PRs

Studies suggest that drugs often complain about the “pushy” behaviour of PRs that puts them under pressure to prescribe a certain product from a certain company. They have been reported to have experienced aggressive sale techniques, which they defined as: asking physicians to justify their current prescribing and even direct requests using emotional appeals to the physicians to prescribe a certain medicine as a favour to the PR. Such aggressive marketing methods are criticized by physicians [29, 30, 33].

*“Such approaches could discourage prescribing a representative’s product or seeing a particular representative again. What we don’t like is a drug rep coming in and questioning us, because I don’t think that’s their role or asking us what we do prescribe, and then why. We don’t like that. Some of them can be quite pushy”* [33].

*“In some cases, the representative imposes on the physician to prescribe a certain product. We can prescribe it in rare cases for some diseases. Some representatives say: I have certain amount of medicine in your pharmacy and it's not dispensing, prescribe, just one or two’. This forced us to refuse to meet him again, because he imposes [on] me to prescribe his product for any patient without any reason”* [21].

Studies also indicate that physicians feel obliged to the PR because of previous service they may have provided. Although this is not a direct pressure from the PR, it can still pressure the prescribers indirectly and affect their prescription patterns, which they dislike [21].

Both direct and indirect pressure from PRs are disliked by physicians and can result in the physician deciding to stop seeing that particular PR [29, 30, 33].

### Theme 4.0: Physicians’ perceptions of the effect of PR visits on prescription patterns

Physicians’ perception as to whether or not their prescribing is influenced by PRs is very important since it can determine their attitudes towards PRs [37].

Wang et al. reported that 36% (n = 32) of ophthalmology trainees reported having changed prescribing behaviour based on the information provided by a PRs, 77% (n = 94) stated that they changed prescribing behaviour based on the availability of medicine samples [27].

Research shows, however, that the majority of physicians are convinced that PR visits do not influence their prescribing behaviour [24, 26, 27, 29, 31, 32, 34, 36]. De Ferrari et al. reported that about 88% of study respondents disclosed that they believe receiving gifts or going for company sponsored lunches does not affect their prescribing [9]. All 15 included studies illustrated one common point: that physicians almost always believe that such interactions can influence their colleagues’ prescription patterns but not theirs; Roy et al. has for example, revealed that most physicians usually do not admit having accepted gifts from PRs and even if they do, they usually believe it does not affect their prescribing. Almost all of them, however, claim to know colleagues who accept gifts and whose prescribing behaviour is influenced by this [30, 38].

*“I think drug reps are a good thing…Just because I have a pen with the name of a drug on it, doesn’t mean I’m going to prescribe it”* [33].

The other reason stated by the physicians for their perceived immunity against biased information is the friendship between them and the PR over the years:

*“I think if you see a rep who you know well … it’s the same rep who you’ve seen for several years, they don’t try and pull the wool over your eyes. They know that if they tell you lies you’ll be seeing them again in six months and you’ll find them out”* [33].

On the contrary, some other physicians found this very same social bond to be the main reason their prescribing behaviour being affected; they felt they were sometimes influenced to prescribe a particular medicine due to their long term and continuous relationship with the representative [33].

### Theme 5.0: Reasons to accept/reject PRs

Understanding the reasons behind physicians accepting PRs would be a breakthrough in understanding the overall nature of the interaction between PRs and prescribers [35].

#### 5.1 Reasons for accepting PRs

##### 5.1.1 Sponsorship, gifts

One incentive behind meeting PRs is the sponsorship, gifts and products provided by them.

*“We were building a new surgery and, you know, we needed some sponsorship”* [33].

*“I don’t mind a nice hotel for a weekend. You don’t get many perks unfortunately as a GP, and I don’t see a problem in that”* [33].

*“I work in non-profit…you know [reps] do provide me with pens…[and] somehow my administrator doesn’t want to spend too much money on office supplies”* [34].

##### 5.1.2 Social aspect of the interaction

Friendship and the social bonds made between physicians and PRs over the years has been proven to have a direct effect on the physicians’ decisions on whether or not to accept a PR. Some physicians considered these meetings as a chance for social interaction and as a break from their busy work routine [21, 33].

The social aspect of the interaction is even strong enough that the physicians who have stopped seeing PRs still make exceptions for the ones who are their friends:

*“Some reps I’ve known for donkey’s years and they know all about my life and I know all about their life and you have a chat about things which are totally unrelated to why they came, but it does make life more interesting and you’re probably more likely to actually retain what they came in to tell you if you’ve had a pleasant time talking to them about your kids or something”* [33].

Participants reported that they sometimes accepted the PRs not because they want to hear about new drugs but just to enjoy the social aspect of the visits.

*“Sometimes we don’t even talk about drugs, we just chat about the kids and it’s good to have a relaxed and friendly lunch”* [34].

##### 5.1.3 Courtesy and tradition

Another reason for physicians to agree to meet PRs is the matter of courtesy, where physicians accept to meet a PR just so that they are not rude; some of the sample comments are as follows:

*“I think they have a very difficult job. There will be an element of empathy for somebody who comes and says can I talk to you about something. Out of politeness, really”* [33].

*“I know it’s just the guy’s job, and if I don’t talk to him then he may lose it, so I talk to him”* [33].

*“The other side is to facilitate services for colleagues as they do this task [to support] their families. This refers to a social and economic situation for colleagues because he gets a payoff to spend on his family”* [21].

Some physicians accepted representatives simply because it was tradition or, in other words, part of the job of a physician. Some reported ‘inheriting’ such visits from their previous colleagues, the practice then becoming part of their routine [23].

#### 5.2 Reasons for avoiding/rejecting PRs

Not all physicians readily accept PRs, there are a few physicians who either have a certain criteria for choosing PRs or choose to avoid them completely [23]. The criteria used by some physicians are: personal style, company and the kind of drugs PRs offer.

*“I have specific criteria for selection. I mean, whether I like this representative or not, whether I am comfortable with him or not and whether his style is true or not true. Is he logical or not logical? There are companies that [I] do not care about them. For example, a new company whose products are widely available such as popular products. I often do not meet them because they do not give us new ideas”* [21].

One of the physicians who avoids PRs’ comments*:*

*“Really, from the time that I came here to work, I [have tried] to avoid meeting them because my use is limited, but I have to meet my colleagues. I try to avoid the interview because I know that I will not prescribe his product. I am a surgeon and my use is limited. Just I have painkiller. There is no other choice”* [21].

Physicians cited many reasons for refusing to meet PRs, such as bad experience with PRs and commercial context (e.g., disagreements about some commercial deal), obligations to other companies, lack of conviction about the product, lack of credibility of PRs and workload or inappropriate timing of visits [21, 33].

*“The other thing, I may refuse to meet [a] representative if the owner of the company behaves with our colleagues [in an] inhuman or dishonorable [way], so this forces us to stop prescribing its product and prescribe a similar alternative that exists in the market”* [21].

Reasons for avoiding PRs, other than the ones mentioned above, were: commercially-biased information, pushy and argumentative approach, and a lack of accuracy in the information provided, and, finally, the PR’s influence on prescribing behaviour.

“*I have been very influenced in the past by my prescribing so I don’t see them anymore now. I was getting no advantage from it at all, it was skewing my prescribing and I was losing a lot of time, so I stopped seeing them*” [33].

### Theme 6.0: Guidelines

#### 6.1 Guidelines and their impact

Over the years, there have been a number of guidelines developed by different healthcare societies regarding the interaction between physicians and PRs such as; the American College of Obstetricians and Gynecologists (ACOG), the American Medical Association (AMA) and the Association of American Medical Colleges, Canadian Medical Association (CMA). Morgan et al. [25] and Anderson et al. [28] have each investigated the use and effect of guidelines in the US. Anderson et al. reports that 154 (62%) of participants in the US were familiar with guidelines on interacting with the pharmaceutical industry, of whom 81 (33%) had read guidelines developed by ACOG, 86 (35%) had read AMA guidelines, and 49 (21%) had read guidelines from other sources such as hospital guidelines, journal articles, and continuing medical education programmes [28].

Studies show that those who had read the guidelines would actually provide fewer free samples to patients or have less frequent meals with the PRs. They were also less likely to receive first hand news on medical products from PRs or include PRs in the decision as to whether to prescribe a new drug, compared to those who had not read the guidelines [25, 28].

According to Anderson et al., there was no significant difference between those who reported reading ethical guidelines and those who did not do so pertaining to using PRs for obtaining drug information [28]. It is postulated that having read guidelines did not affect the perceived value of PRs [28].

#### 6.2 Physician’s opinion on guidelines that restrict physician-PR interactions

The opinion of physicians on the policies that limit or prohibit the physician-PR interaction varied significantly. Some participants believed such restrictions to be unfair to prescribers and patients; some welcomed them with open arms. While others reported having doubts about policies, particularly those related to prohibition of free drug samples. These physicians however, disclosed that, over time they realized that their prescribing behaviour had actually been affected by the visits of PRs and availability of free drug samples, and they eventually started supporting policies that prohibit/restrict PR visits. In a study done in the US in 2003, 39.9% of participants stated that they disagreed with placing limitations on the interaction between physicians and PRs, while roughly the same percentage (33.3%) agreed with the policy. In Germany the data is slightly different, over half of the German physicians (n = 108; 52%) indicated that they would regret the cessation of the visits while the remaining 45% (n = 94) supported it [29, 34].

*“I was really hesitant about getting rid of the sample closet years ago, but now I think it was really, definitely the right thing because I would reach for the best non-steroidal that was in there and at that point it was [brand name]. So I give a patient [brand name] thinking I did a good thing because he told me he didn’t have any money, but often they would come back wanting [brand name] where I just could have given him Ibuprofen. …Once we didn’t have it anymore, I realized that…”* [34].

## Discussion

As part of the health care system, pharmaceutical manufacturers have benefited countless people through their investment in research and product development. Their ultimate responsibility, however, is to their shareholders, who expect a reasonable profit from their investments [6]. The pharmaceutical industry, therefore, cannot be entirely blamed for trying to increase returns, within legal boundaries. Inappropriate marketing practices that may arise from this profit-led competition have been proven, however, to have the potential to influence physician prescribing.

Policymakers have therefore tried to restrict the interaction between physicians and PRs, which is where most of the marketing occurs, by developing guidelines and making relevant policies. The World Health Assembly, in an attempt to tackle the issue, adopted the WHO Ethical Criteria for Medicinal Drug Promotion in 1988, which requires PRs to have an appropriate educational background and be adequately trained with sufficient medical and technical knowledge and integrity to present information on products in an accurate, unbiased, and responsible manner. WHO also holds employers responsible, for not only the basic and continuing training of their representatives, but also for their statements and activities [39]. Many critics, however, have complained that these guidelines have been largely disregarded, including the voluntary Code of Pharmaceutical Practices developed by the industry’s own International Federation of Pharmaceutical Manufacturers’ Associations (IFPMA) [1]. While the development of guidelines and policies has proven helpful, therefore, it certainly is not enough..

As demonstrated by this meta-synthesis, the attitude of physicians towards PRs is a very crucial determinant of the potential of PRs to indirectly influence the health of patients in a positive or negative way. The studies included in this meta-synthesis were undertaken in nine different countries with significantly different economies, cultures, education/healthcare systems, and health policies, yet the positive attitude of physicians towards PRs was evident in all of them.

As stated earlier, there are a number of reasons behind this positive attitude with the one-to-one medium of interaction being one of them; the results of the research conducted by McGettigan et al. showed that the information sources most frequently rated important by physicians were not those most used in practice. The sources of practical importance were those involving the transfer of information through personal face-to-face contact, indicating the importance of the medium through which information is conveyed [40] which poses an advantage for direct pharmaceutical detailing.

Lack of time to read and keep abreast of the myriad of new medical information is another important facilitator for physicians to meet PRs. PRs are seen as convenient and timely sources of medicine related information. Physicians perceive the information provided by PRs as factual but in some cases biased. Our findings are in line with that of the systematic review conducted by Wazana et al. [11];physicians believe that they are immune from any potential marketing influence. This is mainly because most of physicians believe that they have the required expertise and knowledge to assess the presented information and distinguish the valid information from the exaggerated, biased information [21, 22, 24–27, 29, 32–34]. This is despite the clear evidence in the literature regarding the effect of PRs on prescribing behaviour [32].

Undeniably, detailing poses as a convenient face-to-face educational meeting [21, 23, 24, 26, 29, 33, 34],especially for newly launched medicines [33, 35]. Instead of seeking to ban these interactions, therefore, medical regulatory bodies could implement proactive measures to educate medical students about potential medical inducements [10]. Vinson et al. measured the effect of a one-hour lecture and discussion about the appropriateness of pharmaceutical gifts among second-year medical students. Findings from the survey have showed that these students had become statistically significantly more resistant to the gifts compared to the first-year students who served as a control group and had not experienced the lecture. This study strongly suggested that changes in students’ attitudes towards marketing methods used by PRs may be fostered [41]. Similarly, positive long term effects were observed from educating trainees and physicians about understanding and responding to pharmaceutical promotion [42–44].

We believe that this meta-synthesis has given new insights into the PR-physician interaction. The findings of this meta-synthesis have implications for policy makers and educators. Future research should focus on practical education and policy interventions to better limit any potential conflict of interest that may arise from such interactions through education and informed policy development.

### Limitations

This meta-synthesis focused on the recent literature and excluded studies published before 2000. This means that some relevant, important points might have been missed if they were published before 2000. Meanwhile, the themes were generated in such a way as to offer, in the authors’ opinion, a useful insight into the different aspects of the PR-physician interaction. Drawing together data from multiple countries has some value, certainly. However, looked at from the opposite point of view, it is not entirely clear that the cultures of pharmaceutical companies and of physicians, and the regulatory/healthcare systems in countries as diverse as Peru, India, the Yemen, the UK and the US can be easily comparable. Direct to consumer advertising is, for example allowed in US but banned in other countries. Thus, there is a need for some further country specific studies.

## Conclusions

The purpose of this meta-synthesis, was to highlight the detailing aspects of PR-physician interactions from the physician’s point of view. This meta-synthesis shows that physicians generally see meetings with PRs as advantageous to everyone: the patients, because they receive free drug samples, the hospital/clinic, because they would receive stationery, books, and, most importantly, themselves, as these meetings help them to stay up-to-date and aware of newly launched medications. Future research should focus on educating medical students to correct their perception of immunity against marketing which may hold them back from critically appraising the information provided by PRs. This will ensure that patients do not bear the cost of competition between pharmaceutical companies.

## Additional files

Additional file 1: Table S1. Description of the search strings used and the results obtained. (DOCX 13 kb)
Additional file 2: Table S2. PRISMA 2009 Checklist. (DOC 209 kb)
Additional file 3: Data extraction from the studies included in the review. (DOCX 130 kb)
Additional file 4: Table S3. Qualitative data demonstrating the physician’s attitude. (DOCX 34 kb)

## Abbreviations

ACOG: American College of Obstetricians and Gynecologists
AMA: American Medical Association
CMA: Association of American Medical Colleges, Canadian Medical Association
GP: General Practitioner
IFPMA: International Federation of Pharmaceutical Manufacturers’ Associations
MO: Medical officer
NA: Not applicable/available
PR: Pharmaceutical representative
SD: Standard deviation
UK: United Kingdom
US: United States
WHO: World Health Organization

## References

[1] WHO. Trade, foreign policy, diplomacy and health. 2014. Available online: http://www.who.int/trade/glossary/story073/en/. Accessed 10 Aug 2014.

[2] Othman N, Vitry AI, Roughead EE, Ismail SB, Omar K (2010). Medicines information provided by pharmaceutical representatives: a comparative study in Australia and Malaysia. BMC Public Health.

[3] Spurling GK, Mansfield PR, Montgomery BD (2010). Information from pharmaceutical companies and the quality, quantity, and cost of physicians’ prescribing: a systematic review. PLoS Med.

[4] IMS Health (2015). Global Pharmaceuticals Marketing Channel Reference.

[5] Gagnon MA, Lexchin J (2008). The cost of pushing pills: a new estimate of pharmaceutical promotion expenditures in the United States. PLoS Med.

[6] Brennan TA, Rothman DJ, Blank L (2006). Health industry practices that create conflicts of interest: a policy proposal for academic medical centers. JAMA.

[7] Stark TJ, Brownell AK, Brager NP, Berg A, Balderston R, Lockyer JM. Exploring Perceptions of Early-Career Psychiatrists About Their Relationships With the Pharmaceutical Industry. Acad Psychiatry. 2015;40:249-54. 10.1007/s40596-015-0403-0 26296632

[8] Stark T (2014). Interactions between physicians and the pharmaceutical industry: A study into the perceptions of the early career psychiatrist.

[9] De Ferrari A, Gentille C, Davalos L, Huayanay L, Malaga G (2014). Attitudes and relationship between physicians and the pharmaceutical industry in a public general hospital in Lima, Peru. PloS One.

[10] Blumenthal D (2004). Doctors and drug companies. N Engl J Med.

[11] Wazana A (2000). Physicians and the pharmaceutical industry: is a gift ever just a gift?. JAMA.

[12] Edwards DA (2000). Gifts to physicians from the pharmaceutical industry. JAMA.

[13] Howard SM (2000). Gifts to physicians from the pharmaceutical industry. JAMA.

[14] McKinney WP, Rich EC (2000). Gifts to physicians from the pharmaceutical industry. JAMA.

[15] Tenery RM (2000). Gifts to physicians from the pharmaceutical industry. JAMA.

[16] Tenery RM (2000). Interactions between physicians and the health care technology industry. JAMA.

[17] Vollmann J (2000). Gifts to physicians from the pharmaceutical industry. JAMA.

[18] Liesegang TJ (2000). Physicians and the pharmaceutical industry: is a gift ever just a gift? Wazana A. JAMA 2000;283:373–380. Am J Ophthalmol.

[19] Green J, Thorogood N (2013). Chapter 10 Reading, Appraising and Integrating Qualitative Research. Qualitative Methods for Health Research.

[20] Walsh D, Downe S (2005). Meta-synthesis method for qualitative research: a literature review. J Adv Nurs.

[21] Al-Areefi MA, Hassali MA, Ibrahim MI (2013). Physicians’ perceptions of medical representative visits in Yemen: a qualitative study. BMC Health Serv Res.

[22] Alssageer MA, Kowalski SR. A survey of pharmaceutical company representative interactions with doctors in Libya. Libyan J Med. 2012;7:18556. http://dx.doi.org/10.3402/ljm.v7i0.18556. 10.3402/ljm.v7i0.18556 PMC3448042 23002397

[23] Brett AS, Burr W, Moloo J (2003). Are gifts from pharmaceutical companies ethically problematic? A survey of physicians. Arch Intern Med.

[24] Misra S, Ganzini L, Keepers G (2010). Psychiatric resident and faculty views on and interactions with the pharmaceutical industry. Acad Psychiatry.

[25] Morgan MA, Dana J, Loewenstein G, Zinberg S, Schulkin J (2006). Interactions of doctors with the pharmaceutical industry. J Med Ethics.

[26] Saito S, Mukohara K, Bito S (2010). Japanese practicing physicians’ relationships with pharmaceutical representatives: a national survey. PLoS One.

[27] Wang Y, Adelman RA (2009). A study of interactions between pharmaceutical representatives and ophthalmology trainees. Am J Ophthalmol.

[28] Anderson BL, Silverman GK, Loewenstein GF, Zinberg S, Schulkin J (2009). Factors associated with physicians’ reliance on pharmaceutical sales representatives. Acad Med.

[29] Lieb K, Brandtönies S (2010). A survey of German physicians in private practice about contacts with pharmaceutical sales representatives. Dtsch Arztebl Int.

[30] Roy N, Madhiwalla N, Pai SA (2007). Drug promotional practices in Mumbai: a qualitative study. Indian J Med Ethics.

[31] Alssageer MA, Kowalski SR (2013). What do Libyan doctors perceive as the benefits, ethical issues and influences of their interactions with pharmaceutical company representatives?. Pan Afr Med J.

[32] Sarikaya O, Civaner M, Vatansever K (2009). Exposure of medical students to pharmaceutical marketing in primary care settings: frequent and influential. Adv Health Sci Educ.

[33] Prosser H, Walley T (2003). Understanding why GPs see pharmaceutical representatives: a qualitative interview study. Br J Gen Pract.

[34] Fischer MA, Keough ME, Baril JL (2009). Prescribers and pharmaceutical representatives: why are we still meeting?. J Gen Intern Med.

[35] Mintzes B, Mangin D, Hayes L and Organization WH. Understanding and responding to pharmaceutical promotion: a practical guide. World Health Organization; 2009. Available from: http://haiweb.org/wp-content/uploads/2015/05/Pharma-Promotion-Guide-English.pdf. Accessed 2 May 2016.

[36] World Health Organization/Health Action International. Understanding and responding to pharmaceutical promotion- a practical guideline. 2010. Available from: http://www1.paho.org/hq/dmdocuments/2011/drugpromotion-manual-CAP-3-090610.pdf. Accessed 10 June 2016.

[37] Theodorou M, Tsiantou V, Pavlakis A (2009). Factors influencing prescribing behaviour of physicians in Greece and Cyprus: results from a questionnaire based survey. BMC Health Serv Res.

[38] Cialdini RB (2009). Influence: Science and practice.

[39] World Health Organisation (1988). Ethical criteria for medicinal drug promotion.

[40] McGettigan P, Golden J, Fryer J, Chan R, Feely J (2001). Prescribers prefer people: The sources of information used by doctors for prescribing suggest that the medium is more important than the message. Br J Clin Pharmacol.

[41] Vinson DC, McCandless B, Hosokawa MC (1993). Medical students’ attitudes toward pharmaceutical marketing: possibilities for change. Fam Med..

[42] King M, Essick C, Bearman P, Ross JS (2013). Medical school gift restriction policies and physician prescribing of newly marketed psychotropic medications: difference-in-differences analysis. BMJ.

[43] Epstein AJ, Busch SH, Busch AB, Asch DA, Barry CL (2013). Does exposure to conflict of interest policies in psychiatry residency affect antidepressant prescribing?. Med Care.

[44] McCormick BB, Tomlinson G, Brill-Edwards P, Detsky AS (2001). Effect of restricting contact between pharmaceutical company representatives and internal medicine residents on posttraining attitudes and behavior. JAMA.

