# Supplementary material for: Interaction and medical inducement between pharmaceutical representatives and physicians: a meta-synthesis
Source: J Pharm Policy Pract. 2016 Nov 17;9:37. doi: 10.1186/s40545-016-0089-z (PMC5114854; doi:10.1186/s40545-016-0089-z)
Supplement: Additional file 1: Table S1. — Description of the search strings used and the results obtained. (DOCX 13 kb) [file 40545_2016_89_MOESM1_ESM.docx]

**S1 Table. Description of the search strings used and the results obtained**

| Search Strings | Database |
| --- | --- |
| ((pharmaceutical representative) AND ethics) AND marketing | PubMed |
| ((medical representative) AND pharmaceutical marketing) AND gifts | PubMed |
| (medical representative and pharmaceutical marketing).mp. [mp=title, abstract, original title, name of substance word, subject heading word, keyword heading word, protocol supplementary concept word, rare disease supplementary concept word, unique identifier] | Ovid Medline |
| (gifts and pharmaceutical representatives).mp. [mp=title, abstract, original title, name of substance word, subject heading word, keyword heading word, protocol supplementary concept word, rare disease supplementary concept word, unique identifier] | Ovid Medline |
| (pharmaceutical representatives and prescriber).mp. [mp=title, abstract, original title, name of substance word, subject heading word, keyword heading word, protocol supplementary concept word, rare disease supplementary concept word, unique identifier] | Ovid Medline |
| all(pharmaceutical marketing) AND all((medical representative OR pharmaceutical representative)) AND all((prescription behavior OR prescription pattern)) | ProQuest |
| all(marketing ethics) AND all((medical representative OR pharmaceutical representative)) AND all((Doctor OR physician)) | ProQuest |
