# Supplementary material for: Interaction and medical inducement between pharmaceutical representatives and physicians: a meta-synthesis
Source: J Pharm Policy Pract. 2016 Nov 17;9:37. doi: 10.1186/s40545-016-0089-z (PMC5114854; doi:10.1186/s40545-016-0089-z)
Supplement: Additional file 2: Table S2. — PRISMA 2009 Checklist. (DOC 209 kb) [file 40545_2016_89_MOESM2_ESM.doc]

| **S2 Table. PRISMA 2009 Checklist.** | | |  |
| --- | --- | --- | --- |
| **Section/topic** | **#** | **Checklist item** | **Reported on page #** |
| **TITLE** | | |  |
| Title | 1 | Interaction and medical inducement between pharmaceutical representatives and physicians: A meta-synthesis. | 1 |
| **ABSTRACT** | | |  |
| Structured summary | 2 | ***Background*:** It has been proven that the interaction between the pharmaceutical representatives and the physician can directly influence the prescribing behaviour. This systematic review aims to explore the available studies regarding the nature of the interaction that takes place between pharmaceutical representatives and physicians.  ***Methods*:** A systematic search was performed of articles published in peer-reviewed journals from January 2000 to April 2015 using PubMed, ProQuest and OvidMedline.  ***Discussion*:** Undeniably, detailing poses as a convenient face-to-face educational meeting,^1-7^especially for newly launched medicines.^6, 8^ Instead of seeking to ban these interactions, therefore, medical regulatory bodies could implement proactive measures to educate medical students about potential medical inducements.^9^  **Limitations**: Papers published in languages other than English were not included as we did not have the necessary skills to interpret them. Moreover, we focused on the recent literature and excluded studies published before the year 2000.  **Conclusion:** This meta-synthesis shows that physicians generally see meetings with PRs as advantageous to everyone: the patients, because they receive free drug samples, the hospital/clinic, because they would receive stationery, books, and, most importantly, themselves, as these meetings help them to stay up-to-date and aware of newly launched medications. Future research should focus on educating medical students to correct their perception of immunity against marketing which may hold them back from critically appraising the information provided by PRs | 1,2,27 |
| **INTRODUCTION** | | |  |
| Rationale | 3 | It has been proven that the interaction between PRs and the physician can directly influence the latter’s prescribing behaviour, leading to increasing concern over irrational and inappropriate prescribing practices ^10^. Given the above, it is crucial to understand the prescriber-focused detailing methods used by PRs. | 3 |
| Objectives | 4 | The purpose of this meta-synthesis, was to highlight the detailing aspects of PR-physician interactions from the physician’s point of view. | 3,4 |
| **METHODS** | | |  |
| Protocol and registration | 5 | Not available |  |
| Eligibility criteria | 6 | The inclusion criteria were articles written in the English language focusing on detailing and physician interaction with PR. Studies related to marketing methods other than detailing such as direct to consumer advertising and ghost writing were excluded. | 5 |
| Information sources | 7 | A search for published articles was conducted in April 2015. Three databases (Pubmed®, Ovid Medline® and ProQuest®) were searched for articles published between January 2000 and April 2015. | 4 |
| Search | 8 | PubMed: ((pharmaceutical representative) AND ethics) AND marketing. | 4 |
| Study selection | 9 | The title and abstract of the papers were assessed for eligibility against the inclusion criteria. This was performed independently by two reviewers, who classified the papers into three groups of “to include”, “to exclude” and “unclear”. In the case of disagreement, or when either reviewer was unsure, the opinion of a third person (LCM) was sought. | 5 |
| Data collection process | 10 | The full text of papers was read by the first author, the non-English language studies (that had their title and abstract written in English and were hence not detected earlier) or those with an irrelevant focus of the study were excluded.The included papers were then subjected to data extraction using a pre-designed data-extraction Excel worksheet (Microsoft Office 2014). | 5 |
| Data items | 11 | **Theme 1.0:PRs Frequency of visits:**  **Theme 2.0: Perceived ethical acceptability of the PRs- physician interactions:**  **Theme 3.0: Physicians’ attitude towards PR visits:**  3.1- Perceived legitimacy of the PR  3.2- Perceived benefits of the interaction:  3.2.1-Easy access to information  3.2.2-Free gifts and drug samples  3.2.3- Social aspects of the interaction  3.3-Perceived drawbacks:  3.3.1- Negative impact on the patient:  3.3.2- Pressure from PRs:  **Theme 4.0: Doctors’ perceptions of the effect of PR visits on prescription patterns:**  **Theme 5.0: Reasons to accept/reject PRs:**  5.1: Reasons for accepting PRs:  *5.1.1: Sponsorship, gifts:*  5.1.2-Social aspect of the interaction:  5.1.3- Courtesy and tradition  5.2-Reasons for avoiding/rejecting PRs:  **Theme 6.0: Guidelines:**  6.1-Guidelines and their impact:  6.2-Opinions on guidelines that restrict physician-PR interactions: |  |
| Risk of bias in individual studies | 12 | It was not performed in this study because we had also included longitudinal studies and descriptive studies. Furthermore, we did not perform meta-analysis for this study |  |
| Summary measures | 13 | Not applicable because we did not perform meta-analysis for this study |  |
| Synthesis of results | 14 | Not applicable because we did not perform meta-analysis for this study |  |

Page 1 of 2

| **Section/topic** | **#** | **Checklist item** | **Reported on page #** |
| --- | --- | --- | --- |
| Risk of bias across studies | 15 | Not applicable because we do not perform meta-analysis for this study |  |
| Additional analyses | 16 | Not applicable because we do not perform meta-analysis for this study |  |
| **RESULTS** | | |  |
| Study selection | 17 | 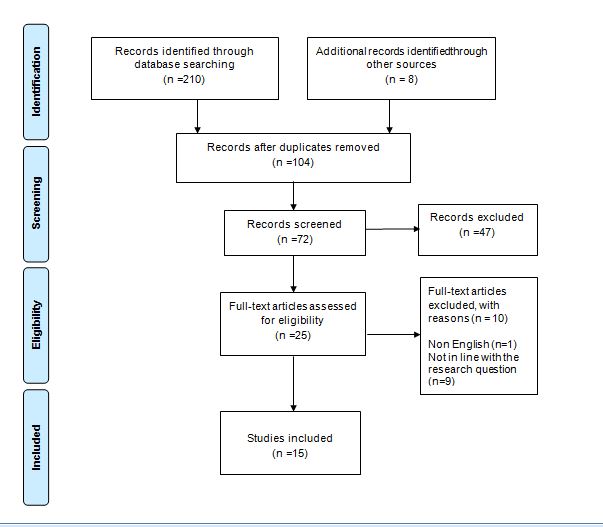 |  |
| Study characteristics | 18 | | **author** | **Journal** | **objective** | **country** | **Population** | **Number of respondents** | | --- | --- | --- | --- | --- | --- | | Sarikaya, O.  Civaner, M.  Vatansever, K. | Advances in health sciences education | This study examines the frequency and influence of student exposure to drug marketing in primary care settings, as well as student perceptions of physician–pharmaceutical company relationships. | Turkey | Third-year students from two medical schools. | 308 | | Wang, Y.  Adelman, R. A. | American Journal of Ophthalmology | To evaluate the behavior and attitudes among ophthalmology trainees toward pharmaceutical promotions. | USA | ophthalmology residents in the United States | 122 | | Misra, S.  Ganzini, L.  Keepers, G. | Academy psychiatric | The authors surveyed psychiatric residents and faculty members on their views and interactions with representatives of the pharmaceutical industry. | USA | psychiatric residents and faculty members | 58 | | Saito, S.  Mukohara, K.  Bito, S. | Plos One | to assess the extent of their involvement in pharmaceutical promotional activities, physician characteristics that predict such involvement, attitudes toward relationships with PRs, correlations between the extent of involvement and attitudes, and differences in the extent of involvement according to self-reported prescribing behaviors. | Japan | Physicians in 7 different specialties | 1411 | | Alssageer, M. A.  Kowalski, S. R. | [Pan Afr Med J](http://www.ncbi.nlm.nih.gov/pubmed/23734277) | to examine perceptions of Libyan doctors between August and October 2010, regarding the benefits, ethical issues and influences of their interactions with (PCRs). | Libya | Libyan Drs | 608 | | Nobhojit Roy, Neha Madhiwalla, Sanjay A Pai | Indian journal of medical ethics | to determine the range of promotional practices influencing drug usage in Mumbai. | India | 15 senior executives in drug companies, 25 chemists and 25 doctors and 36 medical representatives. | 101 | | Mahmoud Abdullah Al-Areefi, Mohamed Azmi Hassali, and Mohamed Izham b Mohamed Ibrahim | BMC Health Services Research | This study aims to qualitatively explore physicians’ attitudes about interactions with medical representatives and their reasons for accepting the medical representatives’ visits. | Yemen | physicians from both private and public hospitals were interviewed this included intern, GPs/medical officers, residents and specialists. 4 females, more than 50% public hospitals | 32 | | Klaus Lieb Simone Brandtönies | Deutsches Arzteblatt International | to assess the kind of contacts that take place and their quality with a survey of physicians in private practice. | Germany | physicians in each of three specialties (neurology/psychiatry, general medicine, and cardiology) | 300 | | Melissa A. Fischer, Mary Ellen Keough, Jerry H. Gurwitz, | J General internal Medicine | To describe the reasons that prescribers from various health professions continue to interact with PRs despite growing evidence of the influence of these interactions. | USA | prescribers (NP, PA, PharmD, MD) | 61 | | De Ferrari, A.  Gentille, C.  Davalos, L.  Huayanay, L.  Malaga, G. | Plos One | To describe physician interactions with and attitudes towards the pharmaceutical industry in a public general hospital in Lima, Peru. | Peru | Physicians | 148 | | Alssageer, M. A.  Kowalski, S. R. | Libyan J Med | To examine the frequency of pharmaceutical company representative (PCR) interactions with doctors in Libya and review possible associations between these interactions | Libya | Physicians | 608 | | Allan S. Brett  Wayne Burr  Jamaluddin Moloo | Internal medicines | sought to determine the degree to which physicians regarded common pharmaceutical marketing activities as ethically problematic, and to compare the views of experienced physicians and physicians-in-training. | USA | residents and faculty members | 76 | | Morgan, M. A.  Dana, J.  Loewenstein, G.  Zinberg, S.  Schulkin, J. | J med Ethics | To assess the opinions and practice patterns of obstetrician-gynaecologists on acceptance and use of free drug samples and other incentive items from pharmaceutical representatives | USA | Gynaecologists | 397 | | Helen Prosser Tom Walley | British journal of general practice | To explore GPs, reasons for receiving visits from pharmaceutical representatives. | UK | GPs | 107 | | Anderson, Britta L.  Silverman, Gabriel K.  Loewenstein, George F.  Zinberg, Stanley  Schulkin, Jay | British journal of general practice | To examine relationships between pharmaceutical representatives and obstetrician-gynecologists and identify factors associated with self-reported reliance on representatives when making prescribing decisions. | USA | Gynaecologists | 251 | |  |
| Risk of bias within studies | 19 | Not applicable |  |
| Results of individual studies | 20 | Not applicable |  |
| Synthesis of results | 21 | Not applicable |  |
| Risk of bias across studies | 22 | Not applicable |  |
| Additional analysis | 23 | Not applicable |  |
| **DISCUSSION** | | |  |
| Summary of evidence | 24 | The studies included in this meta-synthesis were undertaken in nine different countries with significantly different economies, cultures, education, healthcare systems, and health policies, yet the positive attitude of physicians towards PRs was evident in all of them. Physicians see meetings with PRs as very advantageous to everyone: the patients, because they receive free drug samples, the hospital or the clinic, because they would receive stationery, books, and, most importantly, themselves, as these meeting help them to stay up-to-date and aware of newly launched medications.  Lack of time to read and keep abreast of the myriad of new medical information is another important facilitator for physicians to meet PRs. PRs are seen as convenient and timely sources of latest medical information. Physicians perceive the information provided by PRs as factual but in some cases biased, however they believe that their knowledge and expertise immunizes them from any potential marketing influence. This is despite the clear evidence in the literature that shows the effect of PRs on prescription behaviour.^11^  Undeniably, detailing poses as a convenient face-to-face educational meeting,^1-7^especially for newly launched medicines.^6, 8^ Future research should focus on educating medical students to correct their perception of immunity against marketing which may hold them back from critically appraising the information provided by PRs. This will ensure that patients do not bear the cost of competition between pharmaceutical companies. | 6,7,8,9,10,11,  12,13,14,15,  16,17,18,19,  20,21,22,23 |
| Limitations | 25 | Papers published in languages other than English were not included as we did not have the necessary skills to interpret them. Moreover, as older studies might not be relevant to current practices, we focused on the recent literature and excluded studies published before the year 2000. Finally, the themes were generated in a way which we felt would offer a useful way to look at the different aspects of the PR-Physician interaction. They are based on our own interpretation of the field, however, and cannot be considered to be the last word. | 27 |
| Conclusions | 26 | The purpose of this meta-synthesis, was to highlight the detailing aspects of PR-physician interactions from the physician’s point of view. This meta-synthesis shows that physicians generally see meetings with PRs as advantageous to everyone. Future research should focus on educating medical students to correct their perception of immunity against marketing which may hold them back from critically appraising the information provided by PRs. This will ensure that patients do not bear the cost of competition between pharmaceutical companies. | 27 |
| **FUNDING** | | |  |
| Funding | 27 | This review received no funding |  |

*From:*  Moher D, Liberati A, Tetzlaff J, Altman DG, The PRISMA Group (2009). Preferred Reporting Items for Systematic Reviews and Meta-Analyses: The PRISMA Statement. PLoS Med 6(6): e1000097. doi:10.1371/journal.pmed1000097

For more information, visit: **www.prisma-statement.org**.

Page 2 of 2

**References**
