# Supplementary material for: Interaction and medical inducement between pharmaceutical representatives and physicians: a meta-synthesis
Source: J Pharm Policy Pract. 2016 Nov 17;9:37. doi: 10.1186/s40545-016-0089-z (PMC5114854; doi:10.1186/s40545-016-0089-z)
Supplement: Additional file 3: — Data extraction from the studies included in the review. (DOCX 130 kb) [file 40545_2016_89_MOESM3_ESM.docx]

**S1File. Data extraction from the studies included in the review.**

**Theme 1.0: The Significance of Pharmaceutical Representative’s visits**

|  | **Study/ Location** | **Findings** |
| --- | --- | --- |
| **1.1: Frequency of the visits** | [1]  GERMANY | 77% (n = 160) of all respondents were visited by PRs at least once a week, and 19% (n = 39) every day. An estimated 15 000 PSR pay 20 million visits to practices and hospitals in Germany every year [1]. |
|  | [2]  YEMEN | The majority of the physicians were routinely visited by MRs. The range of visits was (0–30)/week, with a median (IQR) of 5 (2–13)[2].  Only one of the 32 physicians in the study has never received visits from PRs. |
|  | [3]  TURKEY | 64.0% of the medical students were exposed to direct marketing. (91.2%) were exposed to indirect marketing methods observed as relationships between PCR and physicians. This demonstrates that a student is subjected directly and/or indirectly to different categories of exposure during the process of their primary health care practice [3]. |
|  | [4]  PERU | Ninety five percent of attendings and 85.7% of residents indicated having at least one monthly interaction with PRs.  In these encounters, 91.2% of participating physicians received medical samples and 87.8% promotional material [4].  Anesthesiologists reported the least number of encounters with PRs. pediatricians reported the largest number of monthly encounters with industry representatives [4]. |
|  | [5]  LIBYA | Most physicians (574; 94%) reported that they had been visited by PCRs at least ‘once’ in the last year. Of the 574 doctors, 286 (50%) reported at least one visit a month. Approximately one-fifth of respondents (118; 20.5%) reported that they had been visited at least ‘once a week’, while 14 (2.6%) physicians reported one or more interaction with a PCR each day[5].  Physicians who had been practicing for in excess of 10 years were more than three times as likely as those having ‘1–3 years of practice’ to meet a PCR at least once a week[5].  Twenty-three (67%) participants who worked in the private sector were visited at least once a week compared with 73 (14%) from the public sector [5]. |
|  | [6]  JAPAN | Most physicians met with PRs (98%), received drug samples (85%) and stationery (96%)[6]. |
| **1.2: Significance of role of PRs as a source of information** | [7]  USA | Participants have reported to have prescribed a median of 3 drugs for the first time in the last year (this includes old drugs prescribed for new indications), 57% of which were introduced to physicians through PRs [7]. |
|  | [8]  INDIA | In Mumbai (India) physicians stated that PR are their primary source of information regarding the new drugs, who present such information in a detailed form using flip charts [8]. |

**Theme 2.0: Perceived Ethical Acceptability of the Interactions**

|  | **Study/ Location** | **Findings** |
| --- | --- | --- |
|  | [1]  GERMANY | Pharmaceutical samples, items of office stationery and free lunches were the most commonly received gifts [1]. |
|  | [4]  PERU | Drug samples are the most received gifts followed by free lunch/dinner  The most ethically perceived activities were receiving medical samples (81.8%) and continuing medical education (68.9%) [4]. |
|  | [8]  INDIA | Very few physicians admitted to having accepted gifts; those who did stated that accepting the gift would not influence their prescription pattern. However, almost all of them knew colleagues who accept gifts and whose prescription pattern is being affected by gifts[8].  Some physicians in india justified accepting gifts as a compensation for the time they spent listening to the PR. However most of them felt bad about it and would agree that imposing limitations on the value of gifts is necessary [8]. |
|  | [5]  LIBYA | 69% of the respondents reported that they had been given drug samples. Nearly half of the surveyed respondents (273; 45%) received free samples at least twice during the last 12 months. Simple gifts were the second most common tools used by PCRs during their promotional visits. The majority of respondents (442; 73%) received simple gifts from pharmaceutical companies[5]. |
|  | [7]  USA | In a study done in South Carolina physicians were asked to answer a questionnaire in which included 18 scenarios of interactions between physicians and the PR. Physicians had to rate the scenarios from most ethically appropriate (score=0) to least ethically appropriate (score=5).   According to the findings of the study, physicians make distinctions about ethical appropriateness of gifts of different value, different type, and the extent of biased information conveyed by the method. The recreational gifts were rated to be significantly less ethically appropriate than the educational gifts. More expensive gifts were also perceived to be less ethically appropriate. Regarding gifts of low financial value, N(28%) of the responding residents and N(43%) of faculty rated gifts such as pencils and pads a score of more than zero, a similar stats was true for inexpensive textbook gift where, N( 41%) of residents and N(49%) of faculty provided responses other than 0 (not problematic), this suggests that there is at least a slight concern about ethical appropriateness of gifts even when they are cheap[7]. |

**Theme 3.0: Physician’s attitude towards PR visits:**

| **Sub-theme** | **Study/ Location** | **Findings** |
| --- | --- | --- |
| **3.1- Perceived legitimacy of the PR** | [9]  USA | participants indicated that overall, the visits are beneficial [9]. |
|  | [4]  PERU | 75% considered that the information brought up by PRs is “not trustworthy” and 80.3% stated that reps “prioritize the promotion of their products over patients' benefit”. Despite this, 47.6% of all participants stated that the information provided by PRs helps them “learn about new products” and “stay up to date” [4]. |
|  | [8]  INDIA | Side effects were hardly ever mentioned by PRs but despite that, physicians were satisfied with the information provided [8]. |
|  | [2]  YEMEN | Physicians recognize the professional authority of PRs as information providers. For example, they deliver information about medicines’ indications, side effects and contraindications, and offer comparisons between one specific product and another. Some physicians cited scientific discussion about the qualities of drugs as reasons for receiving representative visits. However some of physicians are skeptical and accuse PRs of damaging the ethical reputation of the healthcare profession, ignoring the patient’s benefits and refer to them as problem creators. [2] |
|  | [10]  UK | Respondents regarded the info provided by PRs as very variable and depending on the PR you see. The information was considered factual but biased by GPs[10]. |
|  | [1]  GERMANY | 49% (n = 102) of physicians said that they felt that the PSR had informed them adequately or correctly only occasionally, rarely, or never[1]. |
| **3.2- Perceived benefits of the interaction**  3.2.1-Easy access to information: | [1]  GERMANY | The German physicians mentioned the provision of information as the main benefit of the interaction with PRs, with the offer of educational events in second place, and drug samples in third place [1]. |
|  | [6]  JAPAN | Many physicians valued PRs as a source of information. They perceived that the informational value of PRs was higher for new medications than for well-established ones[6].  The extent of physician involvement in promotional activities was positively correlated with the attitudes that PRs are a valuable source of information and that gifts are appropriate [6]. |
|  | [9]  USA | Easy access to information about new and old drugs. Timeliness and convenience of PR meetings were appealing[9]. |
|  | [10]  UK | The face to face interaction helps physicians remember the information better [10].  Physicians also reported that PRs are useful for obtaining research papers and journal offprints. This is found to be mainly due to insufficient independent scientific sources to convey new pharmacological information, and also due to Dr’s busy schedules which leaves them no time to look for evidence. Thus, representatives were seen as ‘short-cuts’, simplifying the acquisition and evaluation of new product information [10].  GPs regarded representatives as a convenient means of acquiring information. Specifically speaking, PRs were regarded to be helpful in Keeping up to date with new/emerging medicines and future developments and reminding physicians of existing drugs accounts [10].  Respondents regarded the PRs as a good source of information who help them keep up to date. They also appreciated the personal interaction. They indicated that PRs are a very convenient timely information sources[10]. |
|  | [4]  PERU | 75% considered that the information brought up by PRs is “not trustworthy” and 80.3% stated that reps “prioritize the promotion of their products over patients' benefit”. In spite of this, 47.6% of all participants stated that the information provided by PRs helps them “learn about new products” and “stay up to date” [4].  Only 24% of faculty (n=10) and 18% of psychiatry residents (n = 3) believed that PRs provide useful and accurate information on new drugs; 20% of faculty (n=8). |
|  | [11]  USA | 12% of residents (n = 2) agreed that representatives provided useful and accurate information on established drugs [11]. |
| 3.2.2-Free Gifts and drug samples: | [11]  USA | The most commonly accepted gifts for both psychiatry faculty and residents were pens, books, and drug samples [11]. |
|  | [12]  LIBYA | Most respondents (n=423; 86%) reported that they had been given printed material (n=480; 79%), simple gifts (stationery, n=442; 73%) or drug samples (n=418; 69%) at least once during the last twelve months[12].  A doctor's attitude towards the acceptance of gifts was significantly associated with the frequency they received printed materials, simple gifts and drug samples . Physicians who had received printed materials and simple gifts materials more than 5 times in the last year were more than three times as likely as those who never received materials to believe it is ethical to accept gifts from PCRs[12]. |
|  | [9]  USA | Some participants talked specifically about PR products that directly benefited patients – educational materials, models, blood sugar diaries [9].  The GPs specifically and very frequently referred to uninsured or indigent patients who would not be able to afford the drug as the main people to benefit from free drug samples while this was in spite the fact that the GS were aware that these patients may not be able to afford the new medication in the long term [9]. |
|  | [4]  PERU | More than 82% of participants were willing to accept medical samples to offer them as free treatment to their resource-poor patients [4]. |
|  | [3]  TURKEY | 52.1% of medical students in 2 universities on Turkey agreed that Physician was giving the samples to the uninsured patients. So the sample drugs were used for a useful purpose[3]. |
|  | [2]  YEMEN | Reception of beneficial patronage or financial support from PRs or even books and stationaries are considered a reason for physicians to accept MRs’ visits[2]. |
|  | [1] | In Germany, Pharmaceutical samples, items of office stationery and free lunches were the most commonly received gifts[1]. |
| 3.2.3- Social aspects of the Interaction | [2]  YEMEN | Social aspect of the interaction is enjoyed by a lot of physicians. Specially in cases where they have known the PR for years  The physicians in Yemen believed that friendship and social interaction are one of the main reasons why physicians accept MRs, they even referred to reception of PRs as their “moral duty”[2]. |
|  | [9]  USA | Some physicians liked the casual, friendly aspect of the interaction and thought of the representatives as more of their friend than a promoter [9]. |
| **3.3-Perceived Drawbacks:**  3.3.1- Negative Impact on the patient: | [1]  GERMANY | German physicians found it disruptive that PRs take up their time [1]. |
|  | [8]  INDIA | India- Physicians stated that PRs took up time that could be spent attending to patients and PRs were aware of this [8]. |
|  | [2]  YEMEN  [9]  USA | Despite the overall positive attitude of the physicians some physicians reported discomfort with or dislike of the interactions, this was rooted in the skepticism of physicians toward the PRs and their perception that PRs harm the reputation of the profession ethically and adversely affect the patients. |
|  | [3]  TURKEY | Some students believed that patients and other healthcare workers are affected negatively by inconvenient PCR practises, that detailing during patient care might hamper the quality of healthcare, and that PCRs are trying to persuade physicians in an unpleasant way[3]. |
| 3.3.2- Pressure from PRs: | [1]  GERMANY | German physicians indicated that they had experienced pushiness from PRs and have been pressured to purchase from PRs, they referred to such methods as disruptive[1].  Germany- 76% (n = 158) stated that PSR often or always wanted to influence their prescribing patterns[1]. |
|  | [2]  LIBYA | Some physicians feel obliged to the PR because of the previous service they have provided.  They also indicated that PRs sometimes visit them not because they want to introduce a certain medicine to them but because they want to make an agreement, they would offer financial support if the physicians agrees to prescribe their product [2]. |
|  | [8]  INDIA | Even PRs are under pressure to meet sales targets [8]. |
|  | [10]  UK | Pushy approach and the use of aggressive marketing methods was not entertained by GPs and were criticized. It also discouraged them from prescribing the certain product[10]. |

**Theme 4.0: Doctor’s perception of the effect of PR visits on prescription pattern.**

| **Sub-theme** | **Study/ Location** | **Findings** |
| --- | --- | --- |
|  | [1]  GERMANY | Only 6% (n = 13) considered themselves to be often or always influenced, while 21% (n = 44) believed this [1, 3, 5, 6, 9-11, 13] of their colleagues[1]. |
|  | [9]  USA | Physicians either trust the information from PRs or feel that they have the expertise to evaluate it independently. |
|  | [4]  PERU | There is a negative relationship between the amount of exposure to industry's promotional activities and reporting that gifts, lunches and other benefits affect prescribing behavior.  The majority of surveyed physicians (88.5%) think that receiving gifts or lunches from industry has no influence on their prescribing behavior, and a smaller proportion (35.2%) think that those same gifts affect their colleagues' prescriptions [4].Attendants were more prone to believe that gifts and lunches do not influence their prescribing behavior (42.2% vs. 23.6%[4]. |
|  | [12]  LIBYA | 80% of the respondents believed that promotional techniques had only a minor effect on their prescription decisions [12]. |
|  | [6]  JAPAN | Physicians believed that their colleagues were more likely to be influenced by promotional activities than themselves.  Japan- Japanese physicians believed that they were unlikely to be influenced by promotional activities, but that their colleagues were more susceptible to such influence than themselves[6]. |
|  | [10]  UK | Physicians were aware of the influence that PR visits have on the prescription pattern [10]. |
|  | [11]  USA | Among the 33 respondents, a significantly higher proportion of respondents believed that gifts did not affect their own prescribing behavior (42%, n= 14), while only 24% (n=8) did not believe that the gifts affected other physicians' behavior [11]. |
|  | [13]  USA | Most Ophthalmology trainees believed that they were less susceptible to influences from pharmaceutical promotions than their peers [13].  Thirty-two trainees (36%) of ophthalmology trainees reported having changed prescribing behavior based on the information provided by a pharmaceutical representative. Ninety-four (77%) stated that they have changed prescribing behavior based on the availability of medicine samples[13]. |
|  | [3]  TURKEY | Direct exposure to marketing methods was most influential in affecting students’ opinions. The odds of students having positive observations or opinions were almost three times higher for those that had been directly exposed to marketing through receiving booklets, gifts, and PC-sponsored meeting invitations than for those who had not been exposed directly[3].  Turkey- Students agreed that physician prescribing behaviours are probably affected by detailing and gifting [3].  Turkey- Direct exposure to PC marketing not only decreases students’ negative opinions about the influences of drug marketing on healthcare, but also engenders positive opinions[3]. |
|  | [2]  YEMEN | Physicians believe that they are immune from being influenced by PRs. In Yemen some physicians accept all PRs while some of them have certain criteria that the PR has to meet before they are allowed to visit the Dr. Some physicians avoid them because they feel PRs are of no help [2]. |

**Theme 5.0: Reasons to accept/reject PRs.**

| **Sub-theme** | **Study/ Location** | **Findings** |
| --- | --- | --- |
| 5.1: Reasons for Accepting PRs:  5.1.1: Sponsorship, gifts: | [9]  USA | Gifts and sponsorships be in it cheap stationeries or expensive ones are one of them main reasons physicians accept to meet a PR[9].  American participants opined that primary care office budgets are tight, and the supplies and food that PRs brought contributed to smooth functioning of the office [9]. |
|  | [12]  LIBYA | The three major perceived benefits reported by physicians from PCR visits were; receiving new information about products (n= 574; 94.4%), invitations to conferences (n=215; 35.4%) and receipt of gifts (n=132; 21.7%)[12]. |
|  | [3]  TURKEY | Medical students reported personal benefits of PC marketing, such as acquiring information through detailing and being rewarded by PCRs[3]. |
| 5.1.2-Social aspect of the interaction: | [2]  YEMEN | Social aspect of the interaction is enjoyed by a lot of physicians. Specially in cases where they have known the PR for years  The physicians in Yemen believed that friendship and social interaction are one of the main reasons why physicians accept MRs, they even referred to reception of PRs as their “moral duty”  Social aspect of the interaction is enjoyed by a lot of physicians. Specially in cases where they have known the PR for years  The physicians in Yemen believed that friendship and social interaction are one of the main reasons why physicians accept MRs, they even referred to reception of PRs as their “moral duty”[2]. |
| 5.1.3- Courtesy: | [9]  USA | Sometimes physicians in USA accept PRs just out of courtesy so that they don’t seem rude[9]. |
|  | [10]  UK | some GPs see PRs as a matter of courtesy. Some did it as a cultural norm because it was simply part of a GPs job and it was tradition[10]. |
| 5.2-Reasons for avoiding/rejecting PRs: | [2]  YEMEN | Some physicians in Yemen reported that the reason they might choose to avoid PRs is the conflict of interest, bad timing of the visit, bad experiences with the PR or with the owner of the company they are representing.Physicians in Yemen who are busy, consider PRs as time wasters and avoid to meet them to be able to attend their patients[2]. |
|  | [10]  UK | Bad timing, PRs lack of knowledge, argumentative approach and influence on prescribing were mentioned as reasons to avoid PRs[10]. |

**Theme 6.0: Guidelines.**

| **Sub-theme** | **Study/ Location** | **Findings** |
| --- | --- | --- |
| **6.1-Guidelines and their impact.** | [14]  USA | Many healthcare societies have developed guidelines regarding the physician-PR interaction such as the American college of obstetrician and Gynecologists (ACOG), the American medical association (AMA) and the association of American medical colleges [14].  154(62%) of participants in USA were familiar with guidelines on interacting with the pharmaceutical industry, of whom 81(33%) have read guidelines developed by ACOG, 86(35% ) read AMA guidelines, and 49(21%) had read guidelines from other sources such as hospital guidelines, journal articles, and continuing medical education programs. 61% of obstetricians reported that reading guidelines has changed the way they practice[14]. |
|  | [15]  USA | Regarding relationships with the industry, almost two thirds (65.6%) of physicians were familiar with the guidelines developed by ACOG, a third (33.0%) with those developed by the AMA and a quarter (25.6%) with those given by the PhRMA. Those who said they were familiar with guidelines given by the ACOG were more likely to agree that they would probably or almost surely accept the consultantship (51.8%v 32.5%; U = 4017.5; p = 0.005), but not the samples or lunch[15].  No association between familiarity with guidelines given by the ACOG and agreement that interactions should be regulated [15]. |
|  | [12]  LIBYA | 99% of physicians (n=602/608,) had never read any guidelines regarding doctor-PCR interactions [12]. |
| **6.2-Opinions on guidelines that restrict Dr-PR interactions:** | [6]  JAPAN | Physicians whose workplaces banned both meeting with PRs and gifts were less likely to meet with PRs than those whose workplaces without such rules[6]. |
|  | [12]  LIBYA | Over a half of surveyed participants (n=349; 57%) reported that they approved of developing policies for restricting the interactions of PCRs with physicians [12]. |
|  | [1]  GERMANY | 52% (n = 108) of physicians would regret the cessation of PSR visits; 45% (n = 94) would not regret this, and no data were available for 3% (n = 6). Those who would regret it is because of the benefits they believe they get from such interactions such as the provision of information, educational events, and free drug samples. The main consequence of restricting these interactions was lack of alternative sources to retrieve reliable information from; 85% (n = 92) of those who would regret the cessation of visits from PSR could not see any alternative for those visits [1].   Germany- Of those who liked prescribing new drugs, 69% (n = 56) would regret the cessation of PSR visits, and 80% (n = 65) could not think of any alternatives to the visits. Of those who preferred prescribing established medications, 40% (n = 46) would regret the cessation of visits, and 57% (n = 66) could not think of alternatives[1]. |
|  | [9]  USA | 39.9% of participants stated that they disagree with placing limitations on the Dr-PR interaction while a roughly same percentage of them (33.3%) agreed to it [9]. |
|  | [15]  USA | Regarding restricting the DR-PR interaction; (39.9%) disagreed, a third (33.6%) agreed and 26.5% were neutral [15]. |
|  | [3]  TURKEY | 75.9% of medical students in a study done in Turkey agreed that ‘Relationships with pharmaceutical companies should be legally regulated [3]. |

**References**

1. Lieb K, Brandtönies S. (2010) A survey of German physicians in private practice about contacts with pharmaceutical sales representatives. Dtsch Arztebl Int 107:392-398.

2. Al-Areefi MA, Hassali MA, Ibrahim MI. (2013) Physicians' perceptions of medical representative visits in Yemen: a qualitative study. BMC Health Serv Res 13:331.

3. Sarikaya O, Civaner M, Vatansever K. (2009) Exposure of medical students to pharmaceutical marketing in primary care settings: frequent and influential. Adv Health Sci Educ 14:713-724.

4. De Ferrari A, Gentille C, Davalos L, Huayanay L, Malaga G. (2014) Attitudes and relationship between physicians and the pharmaceutical industry in a public general hospital in Lima, Peru. PLoS One 9:e100114.

5. Alssageer MA, Kowalski SR. (2012) A survey of pharmaceutical company representative interactions with doctors in Libya. Libyan J Med 7.

6. Saito S, Mukohara K, Bito S. (2010) Japanese practicing physicians' relationships with pharmaceutical representatives: a national survey. PLoS One 5:e12193.

7. Brett AS, Burr W, Moloo J. (2003) Are gifts from pharmaceutical companies ethically problematic? A survey of physicians. Arch Intern Med 163:2213-2218.

8. Roy N, Madhiwalla N, Pai SA. (2007) Drug promotional practices in Mumbai: a qualitative study. Indian J Med Ethics 4:57-61.

9. Fischer MA, Keough ME, Baril JL, Saccoccio L, Mazor KM, Ladd E, et al. (2009) Prescribers and pharmaceutical representatives: why are we still meeting? J Gen Intern Med 24:795-801.

10. Prosser H, Walley T. (2003) Understanding why GPs see pharmaceutical representatives: a qualitative interview study. Br J Gen Pract 53:305-311.

11. Misra S, Ganzini L, Keepers G. (2010) Psychiatric resident and faculty views on and interactions with the pharmaceutical industry. Acad Psychiatry 34:102-108.

12. Alssageer MA, Kowalski SR. (2013) What do Libyan doctors perceive as the benefits, ethical issues and influences of their interactions with pharmaceutical company representatives? Pan Afr Med J 14:132.

13. Wang Y, Adelman RA. (2009) A study of interactions between pharmaceutical representatives and ophthalmology trainees. Am J Ophthalmol 148:619-622.e613.

14. Anderson BL, Silverman GK, Loewenstein GF, Zinberg S, Schulkin J. (2009) Factors associated with physicians' reliance on pharmaceutical sales representatives. Acad Med 84:994-1002.

15. Morgan MA, Dana J, Loewenstein G, Zinberg S, Schulkin J. (2006) Interactions of doctors with the pharmaceutical industry. J Med Ethics 32:559-563.
