# Supplementary material for: Interaction and medical inducement between pharmaceutical representatives and physicians: a meta-synthesis
Source: J Pharm Policy Pract. 2016 Nov 17;9:37. doi: 10.1186/s40545-016-0089-z (PMC5114854; doi:10.1186/s40545-016-0089-z)
Supplement: Additional file 4: Table S3. — Qualitative data demonstrating the physician’s attitude. (DOCX 34 kb) [file 40545_2016_89_MOESM4_ESM.docx]

**S3Table.Qualitative data demonstrating the physician’s attitude.**

| **Study Title** | **Quote** |
| --- | --- |
| **Drug Promotional practices in Mumbai^1^**  **Roy et al., 2007** | "These flipcharts show the benefits of their drugs over the drugs of other companies. They also provide results of studies carried out by them on the drug's efficacy."^1^  "MRs never try to bribe to sell their drug. (Gifts) are just a gesture to say thanks for the time the doctor gives. Let's say a doctor sees three patients in 15 minutes, the MR is costing him those three patients in his 15-minute talk. So the MR tries to compensate with gifts since obviously he can't compensate in cash."  "(Accepting gifts) is unethical because many doctors fall prey to these gimmicks and eventually it's the patient who bears the cost."^1^  "Everything is told in a precise way... medical representatives are well versed with their products and quite capable of answering the doctor's questions.”  "Doctors always perceive MRs' visits as an intrusion. Every minute taken up by the MR is time which could have been spent seeing patients and making money in the clinic. Often, MRs queue up early in the morning for doctors who allow only the first three MRs to see them."^1^ |
| **Understanding why GPs see pharmaceutical representatives: a qualitative interview study**  **Prosser et al.,2003** | “If it wasn’t for the reps, all we’d have about Vioxx is two or three letters from the rheumatologist saying:“By the way, this new anti-inflammatory is OK for people with upset stomachs.” That would be all we’d know, or a bit from MIMS, because there isn’t any other mechanism, so if it wasn’t for the drug reps we’d be left high and dry.”^2^  “Although you try and keep up with journals and such like that, some things go by, you do miss things. So I feel like I’m keeping up to date a little bit. If I didn’t see reps I feel that I would be slightly disadvantaged in terms of my awareness of medications coming through.”^2^  “ I think the answer is it’s user friendly, it’s very user friendly and its easy listening, you know, with your coffee listening to what they’ve got to say.’ several GPs commented that they retained information better when it was communicated verbally”^2^  “I’m sure you could manage if you didn’t see another drug rep and I’m sure you could get the information if you wanted to, it’s just that it’s not that accessible, and it’s also whether you would have the time to actually sit and read it.”^2^  “Such approaches could discourage prescribing a representative’s product or seeing a particular representative again. What we don’t like is a drug rep coming in and questioning us, because I don’t think that’s their role or asking us what we do prescribe, and then why. We don’t like that. Some of them can be quite pushy.”  “I think drug reps are a good thing. The information is useful and they provide a good lunch. Just because I have a pen with the name of a drug on it, doesn’t mean I’m going to prescribe it.” ^2^  “I think if you see a rep who you know well ... it’s the same rep who you’ve seen for several years, they don’t try and pull the wool over your eyes. They know that if they tell you lies you’ll be seeing them again in six months and you’ll find them out.”^2^  “Reps are incredibly influential, whoever walks through the door, their products tend to stick in the back of your mind … It’s subliminal you know, it’s amazing how important they are, and then you say “Oh I’ll give that a go”^2^  “We were building a new surgery and, you know, we needed some sponsorship.”  “I don’t mind a nice hotel for a weekend. You don’t get many perks unfortunately as a GP, and I don’t see a problem in that.”^2^  “This is a confession, really, it’s usually when I have responded to some invitation for them to bring a gift of some sort and I know that having done so, you know, I know that if they’re bringing me a fire extinguisher or something, I know I have no obligation to see them but I, generally speaking, do, so that is the way they get access to me. I suppose it’s bribery.”^2^  “Some reps I’ve known for donkey’s years and they know all about my life and I know all about their life and you have a chat about things which are totally unrelated to why they came, but it does make life more interesting and you’re probably more likely to actually retain what they came in to tell you if you’ve had a pleasant time talking to them about your kids or something.”  “I think they have a very difficult job. There will be an element of empathy for somebody who comes and says can I talk to you about something. Out of politeness, really.”^2^  “I have been very influenced in the past by my prescribing so I don’t see them anymore now. I was getting no advantage from it at all, it was skewing my prescribing and I was losing a lot of time, so I stopped seeing them.”^2^ |
| **Prescribers and pharmaceutical representatives: why are we still meeting?**  **Fischer et al., 2009** | “They help because of the information, the ability to get questions answered, and samples are a big help, especially with indigent populations.”^3^  “We want to make people happy and you make people happy often when you give them a sample.” ^3^  “Going out to dinner as a group….That’s why we do it, more of a social setting outside of the wards.”^3^  “My rule is I [listen but] don’t believe anything they’re saying.”^3^  “We welcome any representative regardless [of] his company, even representatives of Danish companies that have been boycotted, because he is considered as our colleague, so I cannot refuse to meet him. We [have a] discussion and then we choose the suitable medicine for the patient”.^3^  “Representatives help [doctors] a lot and […] provide a lot of support, and his company is famous, so why [would I not] meet him? I mean when the efficiency of the medicine is equal. After that, the representative has an important role to convince the doctor to prescribe his product. For me, I do not reject anyone.”^3^  “I work in non-profit…you know [reps] do provide me with pens…[and] somehow my administrator doesn’t want to spend too much money on office supplies.”^3^  “Sometimes we don’t even talk about drugs, we just chat about the kids and it’s good to have a relaxed and friendly lunch.”^3^  “I know it’s just the guy’s job, and if I don’t talk to him then he may lose it, so I talk to him.”^3^  “I was really hesitant about getting rid of the sample closet years ago, but now I think it was really, definitely the right thing because I would reach for the best non-steroidal that was in there and at that point it was [brand name]. So I give a patient [brand name] thinking I did a good thing because he told me he didn’t have any money, but often they would come back wanting [brand name] where I just could have given him Ibuprofen. …Once we didn’t have it anymore, I realized that…”^3^  “Some of these universities have these big ideas about not letting any drug rep come into their surroundings. Yet, they receive a bazillion grants from drug companies to pay for all these other things that they do.”^3^ |
| **Physicians’ perceptions of medical representative visits in Yemen: a qualitative study** | “[It is positive] that they can inform us about new products […] being launched in the market for the first time. Secondly, we can [hear about] alternatives from other companies that have the same effectiveness, low cost and less side effects.”^3^  “Sometimes we need representatives in providing some medicines that we need it, some books or bulletin. Really, they help us in getting books, CDs and lectures from abroad that provided by some companies. They support us on this side a lot.”^3^  “In some cases, the representative imposes on the physician to prescribe a certain product. We can prescribe it in rare cases for some diseases. Some representatives say: I have certain amount of medicine in your pharmacy and it's not dispensing, prescribe, just one or two’. This forced us to refuse to meet him again, because he imposes [on] me to prescribe his product for any patient without any reason.”^3^  “The other side is to facilitate services for colleagues as they do this task [to support] their families. This refers to a social and economic situation for colleagues because he gets a payoff to spend on his family”.^3^  “I have specific criteria for selection. I mean, whether I like this representative or not, whether I am comfortable with him or not and whether his style is true or not true. Is he logical or not logical? There are companies that [I] do not care about them. For example, a new company whose products are widely available such as popular products. I often do not meet them because they do not give us new ideas.”^3^  “Really, from the time that I came here to work, I [have tried] to avoid meeting them because my use is limited, but I have to meet my colleagues. I try to avoid the interview because I know that I will not prescribe his product. I am a surgeon and my use is limited. Just I have painkiller. There is no other choice”^3^  “The other thing, I may refuse to meet [a] representative if the owner of the company behaves with our colleagues [in an] inhuman or dishonorable [way], so this forces us to stop prescribing its product and prescribe a similar alternative that exists in the market.”^3^ |

1. Roy N, Madhiwalla N and Pai SA. Drug promotional practices in Mumbai: a qualitative study. *Indian journal of medical ethics*. 2007; 4: 57-61.

2. Prosser H and Walley T. Understanding why GPs see pharmaceutical representatives: a qualitative interview study. *The British Journal of General Practice*. 2003; 53: 305-11.

3. Fischer MA, Keough ME, Baril JL, et al. Prescribers and pharmaceutical representatives: why are we still meeting? *Journal of general internal medicine*. 2009; 24: 795-801.
